# Teenage Patients with Class II Subdivision Treated with Aligners and Elastics: A Retrospective Study

**DOI:** 10.3390/medicina60122089

**Published:** 2024-12-20

**Authors:** Vincenzo Quinzi, Andrea Conigliaro, Eda Fani, Lucia Memè, Fabiana Fiasca, Nicolò Carugo, Giuseppe Marzo

**Affiliations:** 1 Department of Life, Health and Environmental Sciences, Postgraduate School of Orthodontics, University of L’Aquila, 67100 L’Aquila, Italy; vincenzo.quinzi@univaq.it (V.Q.); andrea.conigliaro@student.univaq.it (A.C.); eda.fani@graduate.univaq.it (E.F.); fabiana.fiasca@alice.it (F.F.); giuseppe.marzo@univaq.it (G.M.); 2 Department of Clinical Sciences and Stomatology, Polytechnic University of Marche, 60126 Ancona, Italy; l.meme@staff.univpm.it

**Keywords:** class II, aligners, asymmetry, elastics

## Abstract

*Background and Objectives*: This study aimed to evaluate the outcomes of Class II subdivision teenage patients treated with Invisalign^®^ clear aligners (CAs) and elastics. *Materials and Methods*: A total of 23 individuals aged 14.3 ± 2.5 years were enrolled in this study. The participants were divided into Group 1 (mandibular midline deviation) and Group 2 (maxillary midline deviation). The midline deviation from the facial midline; anteroposterior discrepancy; overjet (OJ), overbite (OB), and Peer Assessment Rating (PAR) scores; upper incisor and lower incisor (L1) positions; and angulation were measured at the beginning (T0) and end (T1) of the orthodontic treatment. *Results*: Group 1 showed significant higher variations in OJ (−2.3 ± 2.3 vs. −0.6 ± 0.8, *p* < 0.001), OB (−2.1 ± 2.3 vs. −1.1 ± 1.4, *p* < 0.001), PAR score (−32.0 ± 11.7 vs. −27.3 ± 13.1, *p* < 0.001), L1-to-mandibular-plane angle (−3.6 ± 7.0 vs. −1.3 ± 3.2, *p* < 0.001), and interincisal angle (10.07 ± 8.7 vs. 5.9 ± 5.3, *p* = 0.007). The midline deviation was the only measurement with higher variation in Group 2. The average distance between the mesiobuccal cusp of the maxillary first molar and the buccal groove of the mandibular first molar was 0.3 ± 0.5 mm. *Conclusions*: A total of 21 patients achieved bilateral Class I (91% success rate) and demonstrated great improvement (72–96%) in PAR scores. Regardless of the etiology of malocclusion, the orthodontic correction of the Class II subdivision with CAs showed high accuracy and predictable results.

## 1. Introduction

In nature, balance and symmetry are easily recognized and appreciated by the human eye [1]. However, facial correspondence between the opposite sides of the sagittal facial plane is not common in the general population; in fact, minor soft tissue facial asymmetry is frequently observed in normal individuals [2]. This condition, known as subclinical asymmetry or relative symmetry, is not usually perceived by individuals. It is often minimal and clinically insignificant [3], and can be considered a norm rather than an exception. However, significant asymmetry can occasionally lead to functional and esthetic problems. In recent years, improving facial esthetics has emerged as a paramount objective in orthodontic treatment; therefore, the correction of dental occlusion is advocated to align harmoniously with facial features [4]. Class II subdivision is one of the most commonly observed types of occlusal asymmetry [5] and may or may not be associated with facial asymmetry.

Several studies have investigated the nature of the Class II subdivision relationship using two- and three-dimensional radiography (orthopantomography/cone beam computed tomography [CBCT]) or facial photography [6,7] to assess whether the etiology of malocclusion is dental or skeletal.

Clinicians often treat patients with asymmetry [8]. Different orthodontic treatments can be performed depending on the nature of the malocclusion, the patient’s preference, and the effects on periodontal health [9,10]. Class II subdivisions stemming from dental issues and maxillary midline deviations can be corrected with asymmetric extractions or distalization in one arch. Malocclusion caused by mandibular asymmetry may require surgery in young adults [11]. Over the years, various methods have been used to treat patients with asymmetries, such as asymmetric extraction, orthodontic distalizers, fixed appliances, temporary anchorage devices (TADs), bionators, extraoral traction with a facebow headgear, and Class II elastics [12,13,14].

This study aimed to evaluate the outcomes of growing teenage patients with Class II subdivisions treated with clear aligners (CAs) and elastics without the use of asymmetric extractions, auxiliaries such as TADs, fixed sectional appliances, or orthodontic distalizers. Our objectives were to (1) compare the occlusal outcomes (midline correction and molar relationship) after orthodontic treatment with aligners in patients with mandibular or maxillary midline off, (2) evaluate the final mandibular and maxillary incisor positions and angulations, and (3) assess the overall quality of orthodontic treatments using the Peer Assessment Rating (PAR) score.

## 2. Materials and Methods

This study was approved by the Ethics Committee of the University of L’Aquila (Document: DR206/2013; 16 July 2013); furthermore, written informed consent was obtained from all participants.

We searched for patients treated in the last 6 years by the same orthodontist to identify eligible participants. A total of 311 potential participants (aged 10–18 years) were identified between June 2018 and April 2024. Among these, 209 patients who had Class I or Class III molar relationship, 58 who did not meet the subdivision inclusion criteria, 19 who had incomplete medical records, and 2 who had premature loss of the posterior permanent teeth were excluded. Ultimately, only 23 patients met the criteria for inclusion in this retrospective study, which accounted for 7.4% of the initial potential participants. Notably, only two of these patients were in the post-pubertal period. The patients were divided into the mandibular midline deviation group and the maxillary midline deviation group (Table 1).

Group 1 comprised patients whose maxillary midlines aligned with the facial midline, but whose mandibular midlines were deviated to one side. Group 2 comprised patients whose mandibular midline aligned with the facial midline, but whose maxillary midline deviation exceeded 1 mm.

Two patients exhibited deviation of both midlines to the same side, while one patient showed deviation to the opposite side. These patients were allocated to the group corresponding to their major deviation.

This retrospective study involved 23 participants (15 females and 8 males) aged 14.3 ± 2.5 years with Class II subdivision malocclusion who consecutively started orthodontic treatment with Invisalign^®^ (Align Technology, Inc., Tempe, AZ, USA) from 2018 to 2023. Treatment was provided by the same orthodontist in a private practice setting. The initial and final orthodontic records of each patient were obtained, including intraoral and extraoral pictures, digital study models, panoramic and cephalometric radiographs, cephalometric tracings, and comprehensive chart notes. Digital study models were acquired using an intraoral scanner (iTero Element TM Flex; Align Technology Inc., San Jose, CA, USA).

Patients with syndromes, systemic diseases, cleft lip and palate, premature loss of posterior permanent teeth, or crossbites were excluded. Conversely, patients aged between 10 and 18 years, with complete permanent dentition or late mixed dentition, and with anteroposterior (AP) occlusion discrepancy between the right and left sides of at least half of the cusp were included in this study. Patients with a Class I relationship on the left side and a Class II relationship on the right side as well as those with a full-cusp Class II relationship and half-cusp Class II relationship on the opposite side were also selected.

After selecting the eligible patients for this study, two researchers collected data from the orthodontic records. The patients’ charts were reviewed to assess their initial age (years–months), sex, and treatment time. The mandibular and maxillary central incisor angles from the mandibular and maxillary planes were measured by computerized tracing of the initial and final direct digital cephalometric radiographs using NemoCeph (Nemotec Dental Systems, Madrid, Spain). The interincisal angle and distance between the lower central incisor and the A-pogonion (A-Pog) line were also calculated.

The pre-treatment values were incorporated into the initial digital model uploaded on ClinCheck^®^ 6.0 Pro (Align Technology Inc., San Jose, CA, USA). The post-treatment values were calculated for the final digital cast obtained using a digital scan at the end of the orthodontic treatment. The final scans were also uploaded on Invisalign ClinCheck^®^ 6.0 Pro software. However, no further tooth movements were prescribed to ensure that only the values associated with the real final occlusion and molar relationship were read.

The overjet (OJ) and overbite (OB) relationships were measured using ClinCheck^®^ Pro by calculating the average position of the upper central incisor. The OJ and OB measurement data provided by the ClinCheck^®^ digital treatment planning facility were validated using Geomagic Control X, v.2018.1.1 (Geomagic Inc., Morrisville, NC, USA), the gold standard metrology software system [15].

A virtual caliper, the measurement tool of the Invisalign^®^ Software, was used to calculate the horizontal millimetric difference in molar relationship from Class I. The distance between the mesiobuccal cusp of the maxillary first molar and the buccal groove of the mandibular first molar was measured in each patient. A 0 mm disparity indicated a Class I molar relationship, a positive discrepancy denoted a Class II relationship, and a negative discrepancy denoted a Class III relationship.

All 23 orthodontic treatments investigated in this study were aimed at achieving a Class I molar relationship. CAs and 3/16-inch elastics (size: 3.5, 4.5, or 6 ounce [oz]) placed from the canine to the first molar were the only orthodontic appliances used to achieve molar relationship correction. The final molar discrepancy on the Class II side was measured to evaluate whether molar correction was achieved at the end of orthodontic treatment. Molar targets were considered acceptable if the final models showed an AP discrepancy of 1 mm or less.

The mandibular or maxillary midline deviation from the facial midline was measured using patient photographs, digital study models, and ClinCheck^®^ Pro tools; the diagnostic remarks in the note chart were then cross-referenced with this evaluation. Final midlines coinciding with or comprising up to one-fourth of the width of the lower incisor (L1) were regarded as normal. Posteroanterior cephalometric radiographs were available for only a few patients and were not used.

The upper and lower anterior crowds were calculated using the same virtual caliper utilized for determining the AP discrepancy. The contact point displacement was considered as the shortest distance between the contact points of the mandibular incisors parallel to the occlusal plane. The sum of the five measurements represents the irregularity index value of the case [16].

Two examiners randomly and blindly calculated the PAR score. The average value of each examiner’s score was used to assess the initial and final PAR scores in this retrospective study. If the PAR score differed by >10 points, both examiners repeated the measurements. If a >10-point difference persisted after the second measurement, the examiners re-evaluated the casts together to obtain an unambiguous consensus. The difference between the pre- and post-treatment PAR scores (or point decrease) was used to calculate the change in the PAR score. The reduction was multiplied by 100 and divided by the pre-treatment PAR score to obtain the percentage reduction in the PAR score (%).

Patients in Group 1 were treated using a protocol that included a combination of the following interventions:

1. Asymmetric alternating (night/day) mono- and bilateral Class II elastics. During the day (or for approximately 16 h), the patients were prescribed to wear elastics on both sides. At night, the elastic was only worn on the Class II molar relationship side. When both elastics were worn, the elastic on the Class II relationship side was always stronger than the contralateral one (i.e., a 3.5 oz elastic on the right side and a 6 oz elastic on the left side during the day, with only a 4.5 oz elastic on the left side at night).

2. Interproximal reduction (IPR) was performed in the anterior lower (central incisor up to the maximum mesial surface of the 2nd premolar) contralateral hemiarch to improve alignment (particularly in patients with significant crowding and highly triangular incisor shapes), limit the proclination of the incisors due to the use of Class II elastics, avoid gingival recession [17], and achieve a more precise coincidence of the interincisal midline. That is, when the lower midline deviated to the right, IPR was performed in the left hemiarch to align the interincisal lower midline with the upper one. The maximum amount of IPR was 0.3 mm for each contact point.

3. Mild mesial tipping of the lower arch teeth was performed on the Class II side to achieve a better Class I dental fit. Surgery was considered a potential therapeutic option in two patients in the post-pubertal period [18,19]. However, this alternative was turned down in these patients.

The treatment of Group 2 involved mandibular derotation and asymmetric distalization of one hemiarch to align the upper midline with the face, ultimately achieving a Class I molar relationship. Patients in Group 2 used elastics for class correction following the same indications as those prescribed for Group 1. Additionally, IPR was performed in the upper arch of Group 2, mirroring the procedure outlined for Group 1, to enhance the midline coincidence when a discrepancy persisted despite a molar Class I relationship.

### Statistical Analysis

Descriptive statistics (means and standard deviations) were used to examine the characteristics of the sample; sex was expressed as frequencies and percentages. The homogeneity between Group 1 and Group 2 in terms of sex distribution, mean initial age, and mean time of treatment was verified using the χ^2^ test for nominal variables and Student’s *t*-test for continuous variables, as data were normally distributed (Shapiro–Wilk test).

After log transformation of variables with non-normal distribution (midline deviation, OJ, OB, PAR score, upper incisor-to-maxillary-plane angle, and L1 to A-Pog; normality test: Shapiro–Wilk), the differences between the two groups were assessed using a two-way analysis of variance (ANOVA). In the two-way ANOVA, the measurements were used as dependent variables, while the two factors were time (pre- and post-treatment) and deviation from the facial midline (mandibular or maxillary midline).

Statistical analyses were performed using Stata Statistical Software Release 17 (Stata Corp LP, College Station, TX, USA) [20]. A *p* value of 0.05 or lower was considered significant.

## 3. Results

The average deviation of the mandibular or maxillary midline from the facial midline was 1.7 ± 0.7 mm. Group 1 (mandibular midline deviation) comprised 12 participants who showed skeletal asymmetry and chin point deviation in the frontal view. Conversely, Group 2 (maxillary midline deviation) exhibited minimal to no noticeable facial disproportions. The predominant etiology of asymmetry was dental in nature.

A substantial improvement was observed in the final digital cast in both groups. However, Group 1 demonstrated a significantly greater variation in the following measurements at the end of the observation period compared with Group 2: OJ (−2.3 ± 2.3 vs. −0.6 ± 0.8, *p* < 0.001), OB (−2.1 ± 2.3 vs. −1.1 ± 1.4, *p* < 0.001), PAR score (−32.0 ± 11.7 vs. −27.3 ± 13.1, *p* < 0.001), L1-to-mandibular-plane angle (−3.6 ± 7.0 vs. −1.3 ± 3.2, *p* < 0.001), and interincisal angle (10.07 ± 8.7 vs. 5.9 ± 5.3, *p* = 0.007) (Table 2).

In the mandibular midline deviation group, the facial–dental midline distance reduced from 1.6 ± 0.9 mm to 0.5 ± 0.3 mm. In the maxillary midline deviation group, the reduction was even more pronounced, decreasing from 1.9 ± 0.5 mm to 0.4 ± 0.3 mm. Notably, midline deviation was the only measurement displaying higher variations in Group 2 (−1.4 ± 0.5 vs. −1.1 ± 0.8, *p* < 0.001). In both groups, significant variations (*p* < 0.05) were observed in the midline deviation, OJ, OB, PAR score, L1-to-mandibular-plane angle, and interincisal angle before and after treatment.

All 23 participants were adolescents treated with CAs and elastics. The treatment was aimed at achieving a bilateral molar relationship in Class I. The molar targets were considered achieved if the final models showed an AP discrepancy of 0–1 mm.

**Figure 1:**
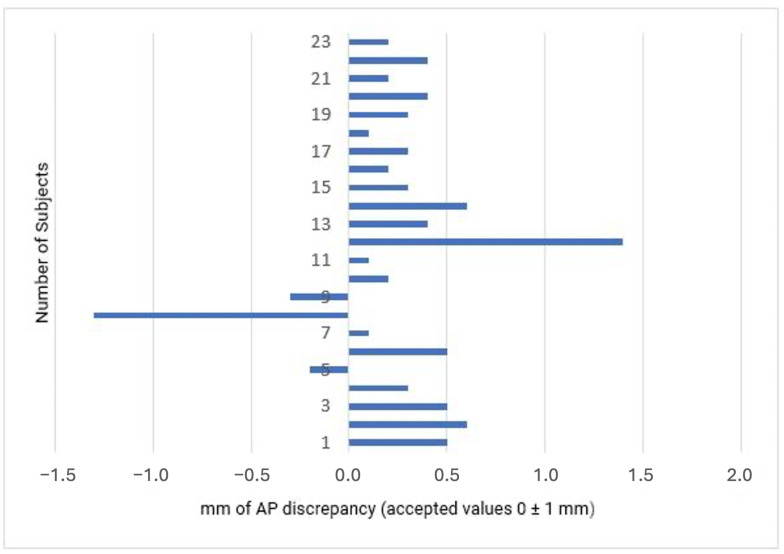
Final molar occlusion observed in 23 patients (position of the mesiobuccal cusp of the maxillary first molar relative to the buccal groove of the mandibular first molar).

Among the 23 patients who completed the orthodontic treatment [21], achieved a Class I molar relationship on the Class II side (91% success rate). One patient demonstrated a super Class I overcorrection, while another patient did not achieve the molar relationship goal of only 0.4 mm from the threshold value, keeping a monoliteral Class II tendency (Figure 1).

Group 1 (mandibular midline deviation) reached the Class I target 83.3% of the time (10 of 12 patients), while Group 2 achieved molar AP discrepancy correction. At the end of the treatment, the buccal groove of the mandibular first molar and the mesiobuccal cusp of the maxillary first molar were separated by an average distance of 0.3 ± 0.5 mm.

**Figure 2:**
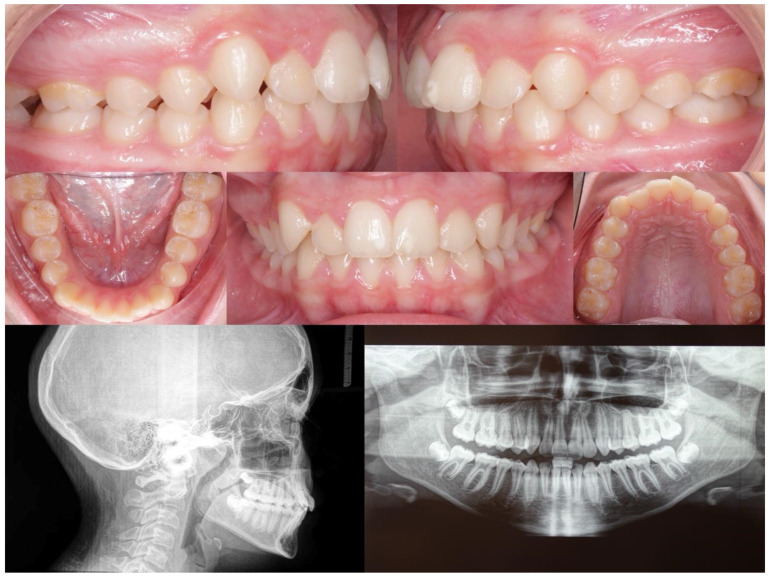
Initial right, left, front, upper, and lower orthopantomography and lateral cephalometric radiographs.

**Figure 3:**
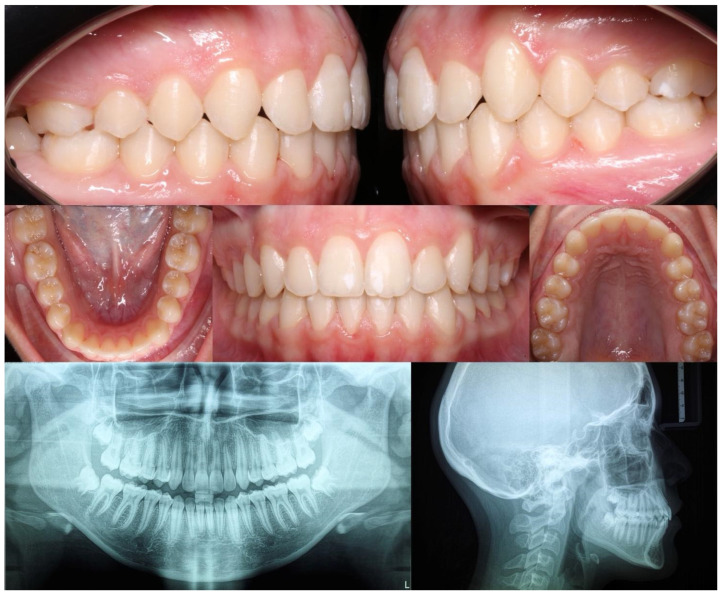
Final right, left, front, upper, and lower orthopantomography and lateral cephalometric radiographs.

Below, the initial (Figure 2) and final (Figure 3) clinical records of a patient with mandibular deviation, treated with the Group 1 protocol, are presented.

## 4. Discussion

This retrospective study assessed the efficacy of digital planning in patients with Class II subdivision receiving orthodontic treatment with CAs. The occlusal outcomes (midline correction and molar relationship), final mandibular and maxillary incisor position and angulation, and overall orthodontic treatment quality were compared based on the PAR scores.

Approximately 32.8% of the study patients had Class II, which was similar to the proportion of patients (36.6%, 95% confidence interval: 33.1–39.5) reported in a survey conducted on 703 schoolchildren aged 12 years old from southern Italy [21]. Moreover, 56.9% of patients with Class II presented with asymmetric malocclusion that could be classified as a Class II subdivision. However, this study highlighted a lower proportion of patients (43.1%) categorized as having a Class II subdivision.

The etiology of Class II subdivision malocclusion can be attributed to skeletal, dental, or a combination of both factors [22,23]. The dental characteristics of Class II subdivision malocclusion include substantial lingual inclination of the mandibular first molar on the Class II side and asymmetry in the sagittal position of the maxillary and mandibular first molars between the two sides [24]. In this study, the patients were deemed to have a skeletal etiology if their mandibular midlines remained asymmetrical after mouth opening, with deviation of the chin toward the Class II side. Conversely, if the midlines coincided, the asymmetry was attributed to a mandibular shift.

The primary contributor to the asymmetric AP relationship in Class II subdivisions with a skeletal etiology is often a shorter and posteriorly positioned mandible on the Class II side [25]. The mandibular midline was not aligned with the facial midline in approximately 61% of these patients [26], which was slightly higher than the 52% observed in our sample. Other studies have highlighted the dentoalveolar component as the primary etiological factor [27,28]. Patients in Group 2 (maxillary midline off) who did not present with mandibular asymmetries were included in this category.

Regardless of the initial etiology, the treatment goal was to achieve bilateral Class I CA, and 3/16-inch elastics (size: 3.5, 4.5, or 6 oz) placed from the canine to the first molar were the only orthodontic appliances used to achieve molar relationship correction. Numerous treatment strategies are available depending on the clinical diagnosis. The ability of a clinician to manage relative growth changes is crucial for the successful treatment of growing individuals [29]. It is essential to adapt clinical protocols according to the needs of individual patients [30].

In our study, 21 of 23 patients who underwent orthodontic treatment had a Class I molar relationship on the Class II side (91% success rate). These results strongly agree with that of a recent case report, which found that Class II and crowding can be effectively treated with aligners and the right auxiliaries in a timeframe equivalent to traditional fixed orthodontic treatment [31].

Molar derotation and asymmetric distalization of one side of the arch toward the center of the upper midline were integral components of the treatment protocol for Group 2. Various studies indicated that 83% of the patients with Class II malocclusion exhibit mesial rotation of the maxillary first permanent molars [32,33]. In addition, the correction of molar rotation allows for more effective molar distalization [34,35]. CAs proved successful in achieving maxillary distal molar rotation, with an observed predictability of 82% and an improvement in molar relationships, despite the challenging nature of this orthodontic movement [36].

Upon completion of the orthodontic treatment, 10 out of 12 patients in Group 1 achieved the Class I goal, translating to an 83.3% success rate. In Group 2, every patient attained a Class I molar relationship. The findings of this study underscore the complexity of midline correction, particularly in patients with mandibular midline deviation. Notably, the midline deviation was the only measurement that displayed higher variations in Group 2 (−1.4 ± 0.5 vs. −1.1 ± 0.8, *p* < 0.001).

Furthermore, our findings demonstrated that patients with the mandibular midline deviation had significantly higher variations in OJ, OB, PAR score, L1-to-mandibular-plane angle, and interincisal angle.

Several studies have evaluated the position and final inclination of the incisors following treatment with CAs. The predicted incisor proclination (69.8%) was partially achieved with CA therapy in patients with Class II subdivision. Factors such as premolar extraction, canine proclination, molar distalization, mini-implants, and patient age affect dental movements [37]. Our results corroborate these assertions, as Group 1 displayed a significant correction in L1 inclination, although normal values were not attained in all participants. Despite this, CAs demonstrated improved control of L1 proclination. This treatment method may be a viable option for treating Class II malocclusions when L1 proclination is undesirable [38].

The final PAR scores significantly increased in both groups and were significant in Group 1 despite the achievement of the Class I relationship in only 83.3% of the patients. Group 1 patients showed more challenging malocclusions, with higher values of OJ, OB, and L1 inclination.

**Figure 4:**
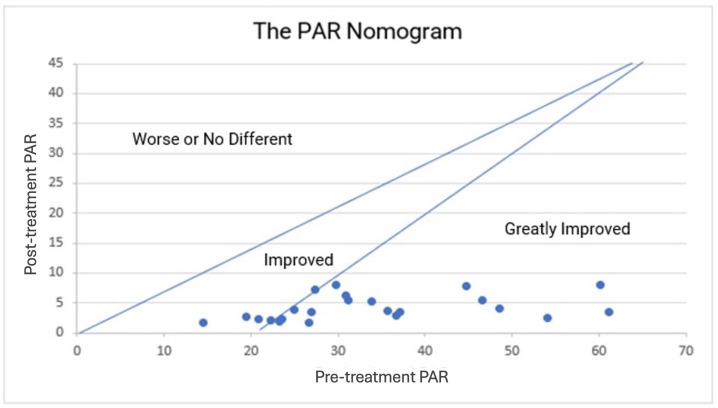
The PAR nomogram: pre-treatment PAR scores are plotted against the post-treatment PAR scores to determine the category of improvement for each patient. PAR, Peer Assessment Rating.

By plotting the PAR score on a nomogram, a two-dimensional graphical diagram that enables the approximate graphical computation of a mathematical function can also yield the results (Figure 4) [39].

In conclusion, our investigation revealed findings that are consistent with those found in the literature, indicating that the complete correction of midlines is not always achievable, particularly in patients with mandibular skeletal asymmetry. Additionally, half of the patients with Class II subdivision exhibited mandibular asymmetry [40].

This study has some limitations. The retrospective design relied on the accuracy and availability of the orthodontic records. AP radiographs and CBCT images were not available to assess the actual skeletal or dentoalveolar asymmetry; only photographs, digital study models, and clinician notes were obtained. Although the number of eligible participants was relatively high, only 23 were included in the final sample.

## 5. Conclusions

Class II subdivisions are prevalent among adolescents. Teenage patients often consider factors related to comfort and quality of life during orthodontic treatment. This patient group may express reluctance toward the use of traditional fixed appliances. Clear alignment with elastics may present a viable alternative for these individuals. Of the 23 patients, 21 achieved bilateral Class I, while all patients demonstrated significant improvements 72–96% in their PAR scores. Regardless of the etiology of malocclusion, orthodontic treatment of the Class II subdivision with CAs showed high accuracy and predictable results.

## Figures and Tables

**Table 1:** Initial characteristics and treatment time.

Variables	Total Sample (n = 23)		Group 1 (n = 12)		Group 2 (n = 11)		*p* Value
Female sex, n (%)	15 (65)		6 (50)		9 (82)		0.110 °
Male sex, n (%)	8 (35)		6 (50)		2 (18)		
	*Mean*	*SD*	*Mean*	*SD*	*Mean*	*SD*	
Initial age (y-m)	14 y 4 m	3 m	14 y 10 m	3 m	13 y 10 m	2 m	0.356 ^#^
Class II side (mm from Class I) *	2.5	0.9	2.8	1.1	2.3	0.5	0.105 ^#^
Class I side (mm from Class I) *	0.6	0.6	0.6	0.5	0.7	0.7	0.481 ^#^
Treatment time (mo)	24.4	7.1	24.2	6.8	24.7	7.7	0.940 ^#^
Values are presented as number (%). Group 1, mandibular midline off; Group 2, maxillary midline off.							
y-m, Years–months							

* These values indicate the position of the mesiobuccal cusp of the maxillary first molar relative to the buccal groove of the manibular first molar. ° χ^2^ test. ^#^ Student’s *t*-test.

**Table 2:** Pre-treatment and post-treatment measurements, stratified by groups.

	Group 1 (n = 12)						Group 2 (n = 11)						
Measurements, Mean ± SD	Pre-Treatment		Post-Treatment		Δ_Group1_		Pre-Treatment		Post-Treatment		Δ_Group2_		*p* Value (Δ_Group1_ vs. Δ_Group2_) *
	Mean	SD	Mean	SD	Mean	SD	Mean	SD	Mean	SD	Mean	SD	
Midline deviation (from facial midline) (mm)	1.6 ^A^	0.9	0.5 ^A^	0.3	−1.1	0.8	1.9 ^B^	0.5	0.4 ^B^	0.3	−1.4	0.5	**<0.001**
Overjet (mm)	4.5 ^A^	2.7	2.2 ^A^	0.7	−2.3	2.3	2.7 ^B^	0.7	2.1 ^B^	0.5	−0.6	0.8	**<0.001**
Overbite (mm)	4.3 ^A^	2.6	2.2 ^A^	0.9	−2.1	2.3	3.3 ^B^	1.2	2.2 ^B^	0.6	−1.1	1.4	**<0.001**
PAR score	35.8 ^A^	13.2	3.8 ^A^	2.3	−32.0	11.7	31.8 ^B^	13.1	4.5 ^B^	2.0	−27.3	13.1	**<0.001**
U1-to-maxillary-plane angle	117.8 ^a^	12.9	111.8 ^a^	5.8	−6.0	14.8	111.1 ^b^	6.7	109.3 ^b^	5.8	−1.8	3.3	0.112
L1-to-mandibular-plane angle	102.4 ^a^	4.4	98.8 ^a^	4.7	−3.6	7.0	93.2 ^b^	4.4	91.9 ^b^	3.7	−1.3	3.2	**<0.001**
L1 to A-Pog(mm)	0.9 ^a^	2.2	0.7 ^a^	2.2	−0.2	2.2	1.3 ^b^	1.4	1.1 ^b^	1.2	−0.2	1.6	0.451
Interincisal angle	124.0 ^A^	9.5	134.7 ^A^	11.0	10.7	8.7	129.7 ^B^	5.7	135.7 ^B^	6.0	5.9	5.3	**0.007**

SD, standard deviation; PAR, Peer Assessment Rating; U1, upper incisor; L1, lower incisor; A-Pog, A-pogonion. * Two-way ANOVA. Bold values denote statistical significance. Group 1, mandibular midline off. T0 vs. T1: a if *p* ≥ 0.05; A if *p* < 0.05. Group 2, maxillary midline off. T0 vs. T1: b if *p* ≥ 0.05; B if *p* < 0.05.

## Data Availability

The raw data supporting the conclusions of this article will be made available by the authors on request.

## Abbreviations

AP	Anteroposterior
CAs	Clear Aligners
CBCT	Cone Beam Computed Tomography
IPR	Interproximal Reduction
TADs	Temporary Anchorage Devices
OPT	Orthopantomography
PAR	Peer Assessment Rating
